# Hepatitis C Virus Network Based Classification of Hepatocellular Cirrhosis and Carcinoma

**DOI:** 10.1371/journal.pone.0034460

**Published:** 2012-04-06

**Authors:** Tao Huang, Junjie Wang, Yu-Dong Cai, Hanry Yu, Kuo-Chen Chou

**Affiliations:** 1 Institute of Systems Biology, Shanghai University, Shanghai, People's Republic of China; 2 Key Laboratory of Systems Biology, Shanghai Institutes for Biological Sciences, Chinese Academy of Sciences, Shanghai, People's Republic of China; 3 Shanghai Center for Bioinformation Technology, Shanghai, People's Republic of China; 4 Graduate School of the Chinese Academy of Sciences, Beijing, People's Republic of China; 5 Centre for Computational Systems Biology, Fudan University, Shanghai, People's Republic of China; 6 Department of Physiology, Yong Loo Lin School of Medicine, National University of Singapore, Singapore, Singapore; 7 Institute of Bioengineering and Nanotechnology, A*STAR, Singapore, Singapore; 8 NUS Graduate School for Integrative Sciences and Engineering, Centre for Life Sciences, Singapore, Singapore; 9 Mechanobiology Institute of Singapore, Temasek Laboratories, National University of Singapore, Singapore, Singapore; 10 Singapore-MIT Alliance, Computational and System Biology Program, Singapore, Singapore; 11 NUS Tissue Engineering Program, DSO Labs, National University of Singapore, Singapore, Singapore; 12 Singapore-MIT Alliance for Research and Technology, Singapore, Singapore; 13 Department of Biological Engineering, Massachusetts Institute of Technology, Cambridge, Massachusetts, United States of America; 14 Gordon Life Science Institute, San Diego, California, United States of America; Saint Louis University, United States of America

## Abstract

Hepatitis C virus (HCV) is a main risk factor for liver cirrhosis and hepatocellular carcinoma, particularly to those patients with chronic liver disease or injury. The similar etiology leads to a high correlation of the patients suffering from the disease of liver cirrhosis with those suffering from the disease of hepatocellular carcinoma. However, the biological mechanism for the relationship between these two kinds of diseases is not clear. The present study was initiated in an attempt to investigate into the HCV infection protein network, in hopes to find good biomarkers for diagnosing the two diseases as well as gain insights into their progression mechanisms. To realize this, two potential biomarker pools were defined: (i) the target genes of HCV, and (ii) the between genes on the shortest paths among the target genes of HCV. Meanwhile, a predictor was developed for identifying the liver tissue samples among the following three categories: (i) normal, (ii) cirrhosis, and (iii) hepatocellular carcinoma. Interestingly, it was observed that the identification accuracy was higher with the tissue samples defined by extracting the features from the second biomarker pool than that with the samples defined based on the first biomarker pool. The identification accuracy by the jackknife validation for the between-genes approach was 0.960, indicating that the novel approach holds a quite promising potential in helping find effective biomarkers for diagnosing the liver cirrhosis disease and the hepatocellular carcinoma disease. It may also provide useful insights for in-depth study of the biological mechanisms of HCV-induced cirrhosis and hepatocellular carcinoma.

## Introduction

Hepatitis C virus (HCV) is an important risk factor for liver cirrhosis and hepatocellular carcinoma [1], [2], [3], [4]. The pathogenesis of these diseases is a multi-step process, including hepatocellular damage and apoptosis, wound-healing responses, inflammatory responses, and hepatocellular regeneration [5]. It is also well known that liver cirrhosis has high potential to lead to hepatocellular carcinoma, especially in the case of HCV-induced cirrhosis [6]. Thus, these two diseases are often correlated with each other, and diagnosis of cirrhosis and HCC at early stages remains challenging [7]. The detailed mechanisms of HCV-induced cirrhosis and hepatocellular carcinoma are unknown [4]. Rapid detection of liver cirrhosis or hepatocellular carcinoma will help provide a timely and appropriate treatment so as to enhance the survival rate of the patient [8], [9]. Understanding of the detailed mechanisms of disease progression can help in developing therapeutic strategies. For example, after revealing the roles of vascular endothelial growth factor receptor (VEGFR) and fibroblast growth factor receptor signaling in hepatocellular carcinoma, their inhibitor Brivanib provides a novel therapeutic treatment against hepatocellular carcinoma [10]. To find effective diagnosis methods for cirrhosis and hepatocellular carcinoma and reveal their mechanisms, knowledge of large-scale HCV infection networks from high-throughput experimental techniques is very useful [11], [12], [13]. In the traditional biomarker studies, the selected biomarkers were often quite different for different studies, and only had a very small overlap [14], [15]. Since there was little concordance among the reported markers, it was hard to identify high-quality biomarkers.

In our approach, we defined two potential biomarker pools, which we will refer to as the “target genes” and “between genes”. The target genes were the human genes associated with the HCV proteins. The between genes were the human genes that were on the shortest paths between the target genes in the protein interaction network. Such two sets of genes have strong biological rationales in correlation with the risk factors that cause liver cirrhosis and hepatocellular carcinoma. Utilizing the concrete HCV-human interaction information would help to exclude the false positive markers. Selecting biomarkers from the target genes and the between-genes would not only make them have an intrinsic correlation with liver cirrhosis and hepatocellular carcinoma diagnosis, but also provide useful information for HCV-induced liver transformation. Indeed, we found that the information of the between-genes among the target genes of HCV can be used to better classify the liver cirrhosis and hepatocellular carcinoma samples than the target genes of HCV. These findings suggest that the interactions between the target genes of HCV are more important than the target genes themselves in triggering liver cirrhosis and hepatocellular carcinoma. It was observed by examining the selected biomarkers that some meaningful correlations did exist among liver cirrhosis, hepatocellular carcinoma, and the genes involved in other cellular processes. The biomarkers found in this study may be of use for diagnosing HCV-induced cirrhosis and hepatocellular carcinoma, as well as for revealing their pathogenic mechanisms.

## Methods

According a recent review [16], to develop a useful model or predictor for biological systems, the following procedures were usually needed to consider: (i) benchmark dataset construction or selection; (ii) mathematical formulation for biological samples that can truly reflect their intrinsic correlation with the target to be predicted; (iii) introducing or developing a powerful algorithm (or engine) to operate the prediction; (iv) properly performing cross-validation tests to objectively evaluate the anticipated accuracy of the predictor. Below, let us elaborate how to deal with these procedures.

### Benchmark dataset: gene expression profiles of normal, cirrhotic, and carcinoma liver tissues

The benchmark dataset used in this study contained 124 tissue samples, of which 19 samples were from normal persons, 58 from the cirrhotic patients, and 47 from the hepatocellular carcinoma patients. The corresponding gene expression profiles for the 19 normal, 58 cirrhotic, and 47 hepatocellular carcinoma (HCC) liver tissue samples were from Mas's work [17] at http://www.ncbi.nlm.nih.gov/projects/geo/query/acc.cgi?acc=GSE14323. The data from the two Affymetrix platforms, HG-U133A and HG-U133A 2.0, were combined by means of the R package matchprobes [18]. The Robust Multi-Array (RMA) method was utilized to process the data [19]. Duplicated probes for each gene were averaged and the processed data were normalized with the quantile method [20]. There were a total of 12,936 genes, and their expression levels were measured in the 124 samples. According to the set theory, the benchmark dataset 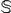 can be formulated as
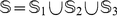
(1)where the subset 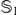 contains 19 normal liver tissue samples, subset 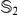 contains 58 cirrhotic liver tissue samples, subset 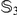 contains 47 hepatocellular carcinoma liver tissue samples, and 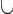 represents the symbol for “union”.

### Tissue sample representation

To develop a powerful statistical prediction method for identifying the attributes of biological samples, one of the most important steps is to extract the core and essential features of the samples that are closely correlated with the target to be identified [21]. According to Eq. 6 of [16], the representation of a tissue sample, or its feature vector, can be formulated as
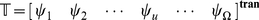
(2)where 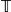 represents the tissue sample, 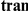 the transpose operator, the components 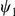, 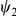, … and 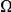 will depend on how to extract the desired information from the tissue sample, as will be elaborated below.

### Hepatitis C virus network

In de Chassey et al.'s study, they identified 481 interactions between HCV and human proteins by the yeast two-hybrid experiments and literature mining [22]. Here, we used the interactions identified by them to construct the hepatitis C virus – human network. The human-protein interaction networks we used were downloaded from STRING [23]. STRING is a comprehensive protein-protein interaction network and the interactions in STRING include physical and functional associations between proteins derived from previous knowledge, genomic context, conserved coexpression and high-throughput experiments [23]. The weight of STRING network was defined as one minus the confidence score.

### The target genes of HCV and the between genes among target genes of HCV

**Figure 1:**
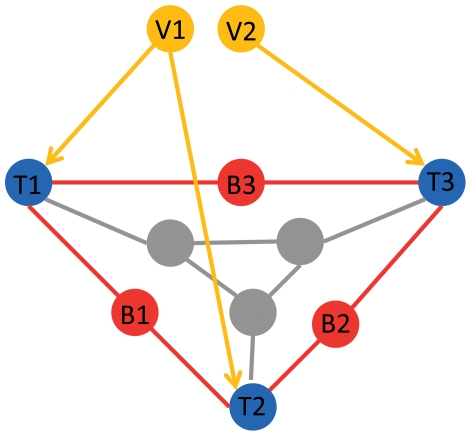
The relationship among the HCV proteins, the target genes and the between genes. The yellow node (V1, V2) are HCV proteins. The target genes (blue nodes, T1, T2 and T3) were the human target genes of HCV proteins. The between genes (red nodes, B1, B2 and B3) were human genes that were on the shortest paths between target genes in protein interaction network. The grey nodes were other human proteins that were neither target genes, nor between genes.

We defined two potential biomarker pools that have strong biological rationales associated with the culprits of the liver cirrhosis and hepatocellular carcinoma: (i) the target genes, and (ii) the between-genes. Figure 1 shows the relationship among the HCV proteins, target genes and the between genes. The target genes were the human target genes of the HCV proteins. The between genes were the human genes that were on the shortest paths between the target genes in the STRING network.

There were 290 target genes associated with the 10 HCV proteins that were measured in our dataset and can be mapped onto the STRING network.

To obtain the between-genes among the target genes of HCV, we linked each pair of the target genes of the 10 HCV proteins by searching the shortest paths between them. The technique we used to find the shortest path was Dijkstra's algorithm [24], [25], [26]. The genes on the shortest paths between the target genes of HCV were defined as the between-genes among the target genes of HCV. There were 684 between-genes among the target genes of HCV.

Accordingly, if using the features of the target genes to represent the tissue samples, Eq. 2 will become a vector with 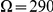 components; i.e.,
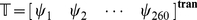
(3)If using the features of the between genes to represent the tissue samples, Eq. 2 will become a vector with 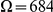 components; i.e.,
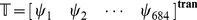
(4)


### Minimum Redundancy Maximum Relevance (mRMR)

In this study, we used the mRMR (Minimum Redundancy Maximum Relevance) approach [27] to select the genes that can be used for classification of liver cirrhosis and hepatocellular carcinoma from the 290 target genes and the 684 between genes, respectively. The advantage of using the mRMR method here is that it can balance the minimum redundancy and the maximum relevance. The maximum relevance would guarantee selecting those features with the most contributions to the classification, while the minimum redundancy would guarantee excluding those features that had already been covered by the selected features. During the selecting process, one feature at a time was selected by mRMR into the selected list. In each round, a feature with the maximum relevance and minimum redundancy was selected. As a result, we obtained an ordered list of features. The mRMR program is available at http://penglab.janelia.org/proj/mRMR/.

### Nearest neighbor algorithm

In this study, the nearest neighbor algorithm (NNA) [28], [29], [30] was used as a prediction engine to identify sample classes as implemented in the NNA program (available at http://pcal.biosino.org/NNA.html). Owing to its good performance and simple-to-use feature, the NNA classifier is quite popular in pattern recognition and has been widely used to deal with varieties of biological problems (see, e.g., [31], [32], [33], [34], [35], [36], [37], [38], [39], [40], [41], [42]). According to the NNA rule, the query sample should be assigned to the same class as the one in the training dataset that is nearest to the query sample. In case there are two or more samples in the training dataset that have exactly the same closest distance to the query sample, then the query sample will be randomly assigned to any one of their classes although this kind of case rarely happens. There are many different metrics to measure the “nearness”, such as Euclidean distance [42], Hamming distance [43], and Mahalanobis distance [44], [45], [46]. In the current study, the following equation was adopted to measure the nearness between two samples:
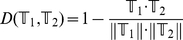
(5)where 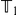 and 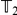 are two vectors representing two samples (cf. **Eq. 1**), 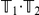 is their dot product, 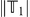 and 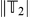 are their moduluses. The smaller the 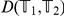, the more similar the two samples are. For a concise formulation of the NNA classifier, see Eq. 17 of [16]; for an intuitive illustration of how the NNA classifier works, see Fig. 5 of [16].

### Jackknife test

In statistical prediction, the following three cross-validation methods are often used to examine a predictor for its effectiveness in practical application: independent dataset test, subsampling test, and jackknife test [43]. However, as illustrated in [47] and demonstrated by Eq. 50 of [31], among the three cross-validation methods, the jackknife test is deemed the least arbitrary that can always yield a unique result for a given benchmark dataset, and hence has been increasingly used by investigators to examine the accuracy of various predictors (see, e.g., [33], [34], [35], [37], [38], [40], [42], [48], [49], [50], [51], [52], [53], [54], [55]. Accordingly, in this study, the prediction model was examined by the jackknife test, also known as leave-one-out cross-validation (LOOCV) test. During the course of jackknife test, each sample in the benchmark dataset was in turn singled out as the prediction target and the rest of the samples were used to train the prediction model. The following equation was used to reflect the prediction accuracy:
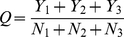
(6)where *Y*_1_, *Y*_2_ and *Y*_3_ represent the numbers of correctly predicted events for the “normal”, “cirrhotic”, and “hepatocellular carcinoma” tissue samples, respectively; while *N*_1_, *N*_2_ and *N*_3_ stand for the numbers of “normal”, “cirrhotic”, and “hepatocellular carcinoma” samples investigated, respectively.

### Incremental feature selection (IFS)

Based on the ranked features according to their importance evaluated by the mRMR approach, we used Incremental Feature Selection (IFS) [56], [57] to determine the optimal number of features. During the IFS procedure, features in the ranked feature set were added one by one from higher to lower rank. A new feature set was composed when one feature had been added. Thus *N* feature sets would be composed for the *N* ranked features. The *i*-th feature set is given by
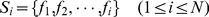
(7)For each of the *N* feature sets, an NNA classifier was constructed and examined using the jackknife test on the benchmark dataset. By doing so we obtained an IFS table with one column for the index *i* and the other columns for the prediction accuracy. Thus, we could obtain the optimal feature set (*S*_optimal_), with which the predictor would yield the highest prediction accuracy.

## Results and Discussion

### The IFS results of target genes and between genes

**Figure 2:**
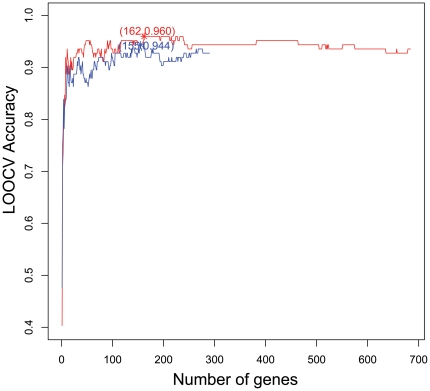
The IFS curves of target genes and between genes. In the IFS curve, the X-axis is the number of genes used for classification, and the Y-axis is the prediction accuracies of nearest neighbor algorithm evaluated by the jackknife test (or LOOCV). The blue curve is the IFS curve of target genes and the highest accuracy was 0.944 with 155 genes. The red curve is the IFS curve of between genes and the highest accuracy was 0.960 with 162 genes.

By analyzing the gene expression profiles for the normal, cirrhotic, and hepatocellular carcinoma liver tissue samples with the mRMR method, we ranked the 290 target genes and 684 between genes according to their importance to liver cirrhosis and hepatocellular carcinoma classification. Subsequently, we selected the optimal gene set from the aforementioned ranked genes by means of the IFS procedure. The IFS curves of the target genes and between genes are shown in **Figure 2**, where the blue curve is the IFS curve for the target genes and the highest accuracy was 0.944 with 155 genes. The red curve is the IFS curve for the between genes and the corresponding highest accuracy was 0.960 with 162 genes. The IFS tables for the target genes and the between genes were given in **Table S1** and **Table S2**, respectively. As shown in **Figure 2**, the accuracies for the between genes were always higher than those for the target genes. The selected 155 target genes and selected 162 between genes can be found in **Table S3** and **Table S4**, respectively. Furthermore, an integrated system containing 916 genes was constructed by combining the set of 290 target genes and the set of 684 between genes. The IFS curve for such 916 target/between genes was shown in **Figure S1**, from which we can see that the corresponding highest accuracy was 0.968 and IFS curve of the combined gen set was twisted with the IFS curve of the between genes, indicating that no significant improvement for the prediction was observed by integrating the target genes with the between genes.

### Analysis of the selected target genes and between genes with HCV

**Figure 3:**
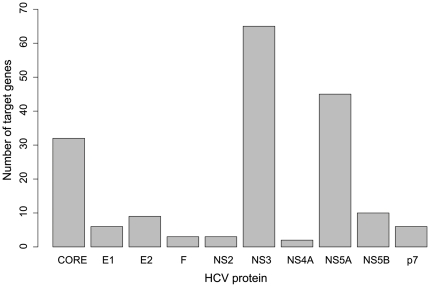
The number of selected target genes of each HCV protein. The HCV proteins NS3, NS5A and CORE have the largest numbers of target genes in the optimal set of the 155 selected target genes.

**Figure 4:**
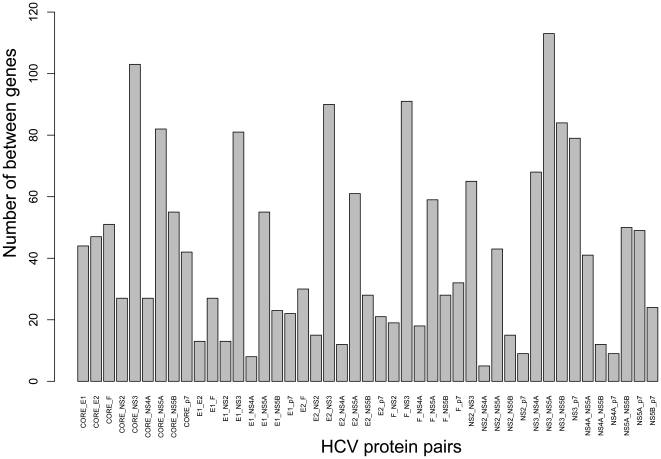
The number of selected between genes for each of the HCV protein pairs. The following pairs have more than 80 selected between genes: NS3_NS5A, CORE_NS3, F_NS3, E2_NS3, NS3_NS5B, CORE_NS5A and E1_NS3.

It is known that HCV is primarily comprised of a single long open-reading-frame encoding an approximately 3000-amino-acid-long protein that is cleaved into three mature structural proteins (CORE, E1, E2), six non-structural proteins (NS2, NS3, NS4A, NS4B, NS5A, NS5B) and a small membrane protein (p7) [58], [59]. To analyze which HCV protein can be related to liver cirrhosis and hepatocellular carcinoma, we calculated the number of the selected target genes for each HCV protein and the number of the selected between genes for each of the HCV protein pairs. Shown in **Figure 3** is the number of selected target genes for each of the HCV proteins. According to **Figure 3**, HCV proteins NS3, NS5A and CORE are the most important ones because they were observed interacting with many target genes in the selected optimal target gene set. The number of the selected between genes for each of the HCV protein pairs is shown in **Figure 4**, from which we can see that the following pairs are involved with more than 80 selected between genes and hence are more important: NS3_NS5A, CORE_NS3, F_NS3, E2_NS3, NS3_NS5B, CORE_NS5A and E1_NS3. Among the above seven pairs, NS3 appeared six times; NS5A, two times; CORE, two times. The outcome is quite similar to that of the target gene. Although there were only 19 genes overlapped between the selected 155 target genes and the selected 162 between genes, the results were quite robust for the HCV protein level. This is because it was found that NS3, NS5A and CORE were important from both the analysis of the selected target genes for each of the HCV proteins and the analysis of the selected between genes for each of the HCV protein pairs. NS3 and NS5A are both non-structural proteins which are responsible for the function of replication and for packaging the viral genome into capsids [58]. NS3 is a bifunctional protease/helicase [60], and is associated with the tumour suppressor p53 [61]. NS3 has been intensely studied as drug targets [62]. Although no enzymatic activity has been ascribed to NS5A, it was reported that an inhibitor of HCV NS5A could suppress virus replication in clinical trials [63]. CORE protein plays an essential role in the formation of virion and it interacts with other HCV proteins [64], [65].

### Comparison of the selected target genes and between genes with the known hepatocellular carcinoma genes

To compare the selected target genes and the between genes with the known hepatocellular carcinoma genes, an enrichment analysis was performed for the 155 selected target genes and the 162 selected between genes on the OncoDB.HCC [66] genes. OncoDB.HCC is a comprehensive database of hepatocellular carcinoma related genes [66]. The results thus obtained for the 155 selected target genes and the 162 selected between genes on OncoDB.HCC genes are shown in **Table 1**, from which we can see that the 162 selected between genes were significantly (having hypergeometric test p value = 1.25E-05) more enriched with the OncoDB.HCC genes than the 155 selected target genes. Besides, the selected between genes also had greater overlapping with the OncoDB.HCC genes in comparison with the 155 selected target genes.

**Table 1:** The enrichment of the 155 selected target genes and the 162 selected between genes on OncoDB.HCC genes.

Gene Set	Hyper geometric test p value	Number of overlapped genes with OncoDB.HCC	Overlapped genes with OncoDB.HCC
Selected target genes	0.001984	15	BAX, CD81, CTGF, FAS, GRN, HSPA5, IGLL1, KRT19, NPM1, RAF1, SERPINF2, SERPING1, SRC, THBS1, VIM
Selected between genes	1.25E-05	20	ALB, AR, CDC20, CDKN2A, COL4A1, CXCL12, DCN, DUSP1, E2F1, ERBB2, GNAS, HSPA5, MAP2K1, MAPRE1, MMP2, MYC, PSMD4, PTK2, ROBO1, SCAMP3

### The biological meanings of the selected target genes and the between genes

To reveal the biological meanings, we performed the KEGG enrichment analysis on the 155 selected target genes and the 162 selected between genes using GeneCodis [67], [68]. Shown in **Table S5** and **Table S6** are the KEGG enrichment results thus obtained for the 155 selected target genes and the 162 selected between genes, respectively. As we can see from the two tables, the 155 selected target genes were enriched on many cancer-related pathways, such as pancreatic cancer, pathways in cancer, chronic myeloid leukemia, colorectal cancer pathways, and other signaling pathways, such as neurotrophin signaling pathway, T cell receptor signaling pathway, B cell receptor signaling pathway, chemokine signaling pathway. Likewise, the 162 selected between genes were also enriched on cancer and signaling pathways, such as pancreatic cancer, chemokine signaling pathway, axon guidance, focal adhesion, and T cell receptor signaling pathway. We also enriched the original 290 target genes and 684 between genes into the KEGG pathways. The selected 155 target genes and selected 162 between genes had more enriched cancer-related pathways and signaling pathways than the original 290 target genes and 684 between genes. Listed in **Table S7** are the numbers of the top 20 enriched KEGG pathways for the 155 selected target genes, the 162 selected between genes, the original 290 target genes, and the original 684 between genes.

The top five genes in the selected target genes were EFEMP1 (EGF-containing fibulin-like extracellular matrix protein 1), JAG2 (Protein jagged-2), TACSTD2 (Tumor-associated calcium signal transducer 2), STAT3 (Signal transducer and activator of transcription 3) and STAT1 (Signal transducer and activator of transcription 1). EFEMP1 binds EGF receptor and activates downstream signaling pathways. Expression of EFEMP1 promotes angiogenesis and accelerates cancer growth [69]. EFEMP1 is a novel tumor-suppressor gene found in hepatocellular carcinoma [70]. JAG2 is involved in the mediation of Notch signaling and is critical for cell development [71], [72], [73]. TACSTD2 encodes a carcinoma-associated antigen and contributes to tumor pathogenesis [74]. STAT3 and STAT1 are members of the STAT (Signal Transducers and Activators of Transcription) family of transcription factors that regulates cell differentiation, growth and survival [75]. In primary tumours, the STAT pathway is usually dysregulated and causes increased angiogenesis, enhanced survival of tumours and immunosuppression [76].

The top five genes in the selected between genes were PDIA3 (Protein disulfide-isomerase A3), LCP2 (Lymphocyte cytosolic protein 2, also known as SLP-76, Src homology 2 domain containing leukocyte protein of 76 kDa), IL23A (Interleukin-23 subunit alpha), SCAMP3 (Secretory carrier-associated membrane protein 3) and ISG15 (Interferon-induced 17 kDa protein). STAT3 ranked sixth in the selected between genes. PDIA3 is part of the MHC (major histocompatibility complex) class I peptide-loading complex, which is vital for the formation of antigen conformation and export from the endoplasmic reticulum (ER) to the cell surface [77]. LCP2 plays important roles in promoting T cell development and activation [78]. IL23A activates the Jak-Stat signaling cascade, induces autoimmune inflammation and may be important for tumorigenesis [79], [80], [81]. SCAMP3 can form association with the EGF Receptor [82]. ISG15 targets to diverse cellular pathways, such as JAK, STAT and MAPK [83] and has antiviral activity [84].

The KEGG enrichment results for the top five target genes (EFEMP1, JAG2, TACSTD2, STAT3 and STAT1) and for the top five between genes (PDIA3, LCP2, IL23A, SCAMP3, ISG15) are given in Table 2, where it can be seen that STAT1 and STAT3 participated in several well-studied hepatocellular carcinoma pathways, such as Jak-STAT signaling pathway, hepatitis C pathway, and pathways in cancer. Interestingly, both the target genes STAT1/STAT3 and the between gene IL23A were involved in Jak-STAT signaling pathway; the latter is associated with HCV clinical syndromes [22], [85].

**Table 2:** The KEGG enrichment of the top five target genes (EFEMP1, JAG2, TACSTD2, STAT3 and STAT1) and the top five between genes (PDIA3, LCP2, IL23A, SCAMP3, ISG15).

KEGG	Corrected hyper geometric p value	Genes
04630 :Jak-STAT signaling pathway	0.000327	STAT1,IL23A,STAT3
05212 :Pancreatic cancer	0.002516	STAT1,STAT3
05160 :Hepatitis C	0.003061	STAT1,STAT3
05162 :Measles	0.003619	STAT1,STAT3
05145 :Toxoplasmosis	0.00439	STAT1,STAT3
04062 :Chemokine signaling pathway	0.004439	STAT1,STAT3
05152 :Tuberculosis	0.004603	STAT1,IL23A
04380 :Osteoclast differentiation	0.005503	LCP2,STAT1
05200 :Pathways in cancer	0.011662	STAT1,STAT3
04330 :Notch signaling pathway	0.033518	JAG2
05140 :Leishmaniasis	0.034105	STAT1
04664 :Fc epsilon RI signaling pathway	0.034608	LCP2
04622 :RIG-I-like receptor signaling pathway	0.036039	ISG15
05221 :Acute myeloid leukemia	0.036899	STAT3
05323 :Rheumatoid arthritis	0.037517	IL23A
04612 :Antigen processing and presentation	0.03827	PDIA3
04620 :Toll-like receptor signaling pathway	0.040087	STAT1
04660 :T cell receptor signaling pathway	0.040175	LCP2
04920 :Adipocytokine signaling pathway	0.040873	STAT3
04650 :Natural killer cell mediated cytotoxicity	0.045794	LCP2

### The advantages of between genes as biomarkers and drug targets

The between genes are not only the coordinator of HCV that triggers the disease-causing signaling, but also the carrier that executes such order and actually causes the pathological changes. Among the top five between genes, ISG15 was on the shortest path of 289 HCV target gene pairs. It regulates and functions in diverse cancer-related pathways [83]. It has been identified as an antiviral molecule [84]. As the bridge of HCV infection, the between genes are responsible for the initiation and progression of hepatocellular cirrhosis and carcinoma. They have closer relationship with the pathological changes during the transformation of hepatocellular cirrhosis and carcinoma than HCV proteins or their target genes. The target genes may indicate the early response of HCV infection, but the between genes can more accurately reflect the post-infection pathological processes and hence be used to serve as a better biomarker. The classification accuracy of the 162 selected between genes was 0.960, higher the accuracy of the 155 selected target genes, 0.944. The accuracy of the top five between genes was 0.815, also higher the accuracy of the top five target genes, 0.782. Classifier based on the between genes performed better than the classifier based on the target genes. Since the between genes play important roles in the course of both initiating the disease and its aggravation, they may become a drug target for both the preventive and therapeutic purposes, like the between gene ISG15 already did [84].

## Supporting Information

Figure S1 The IFS curve of the combined gene set. (A) The IFS curve of the combined gene set, between genes and target genes. The black, red and blue lines represent the IFS curve of the combined gene set, between genes and target genes, respectively. The curve of between genes is consistently higher than the curve of target genes. The curve of combined gene set is twisted with the curve of between genes. (B) The top ten gene IFS curve of the combined gene set, between genes and target genes. The black, red and blue lines represent the IFS curve of the combined gene set, between genes and target genes, respectively. Within the top ten genes, the highest accuracy of between genes is greater than the accuracies of combined gene set and target genes.
(TIF)

Table S1 The IFS table of the target genes.
(XLSX)

Table S2 The IFS table of the between genes.
(XLSX)

Table S3 The selected 155 target genes.
(XLSX)

Table S4 The selected 162 between genes.
(XLSX)

Table S5 The KEGG enrichment result of the 155 selected target genes.
(XLSX)

Table S6 The KEGG enrichment result of the 162 selected between genes.
(XLSX)

Table S7 The top 20 enriched KEGG pathways for the 155 selected target genes, the 162 selected between genes, the original 290 target genes, and the 684 original between genes.
(XLSX)
