# Supplementary figures and images for: Hepatitis C Virus Network Based Classification of Hepatocellular Cirrhosis and Carcinoma
Source: PLoS One. 2012 Apr 6;7(4):e34460. doi: 10.1371/journal.pone.0034460 (PMC3321022; doi:10.1371/journal.pone.0034460)

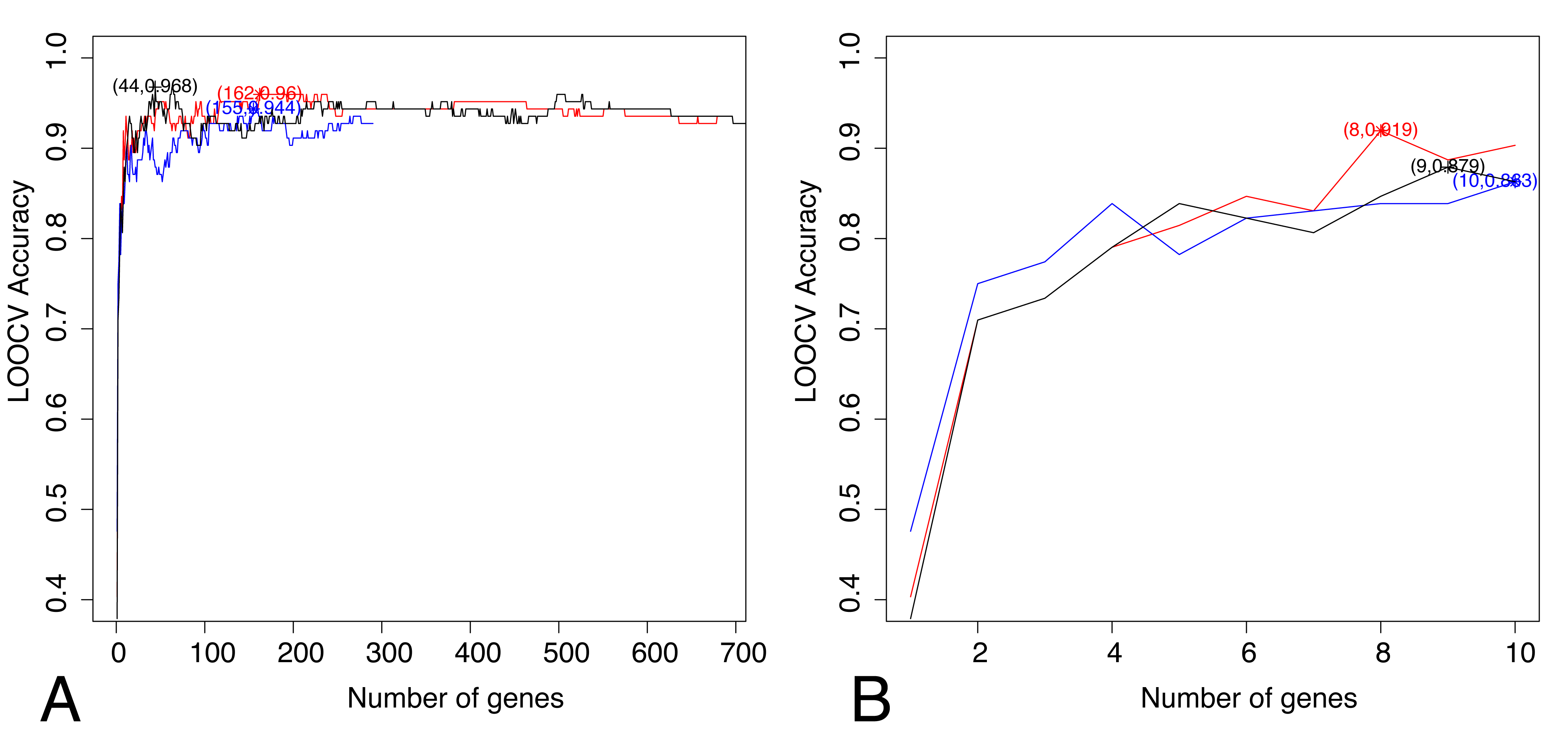

Supplement: Figure S1 — The IFS curve of the combined gene set. (A) The IFS curve of the combined gene set, between genes and target genes. The black, red and blue lines represent the IFS curve of the combined gene set, between genes and target genes, respectively. The curve of between genes is consistently higher than the curve of target genes. The curve of combined gene set is twisted with the curve of between genes. (B) The top ten gene IFS curve of the combined gene set, between genes and target genes. The black, red and blue lines represent the IFS curve of the combined gene set, between genes and target genes, respectively. Within the top ten genes, the highest accuracy of between genes is greater than the accuracies of combined gene set and target genes. (TIF) [file pone.0034460.s001.tif]
